# SHIFT IN ACTIVITY PATTERNS AMONG OLDER ADULTS BEFORE AND AFTER THE COVID-19 PANDEMIC: A SEQUENCE ANALYSIS

**DOI:** 10.1093/geroni/igac059.798

**Published:** 2022-12-20

**Authors:** Wenxuan Huang, Jiao Yu

**Affiliations:** Johns Hopkins University, Baltimore, Maryland, United States; University of Minnesota, Minneapolis, Minnesota, United States

## Abstract

**Background:**

Previous research has discovered significant heterogeneity in older adults’ activity patterns, as well as its associations with well-being. Studying activity pattern during the COVID-19 pandemic sheds new light on infection risks and coping strategies stratified by socioeconomic status. This study answers two research questions: (1) what are the typical activity patterns among older adults in the U.S.? and (2) how do different sociodemographic groups react to the COVID-19 pandemic by alternating activity pattern? Data: This study used data from the American Time Use Survey (2019 and 2020) to create an analytic sample of 5,068 older adults aged 65 and older. Method: First, we classified daily activity sequences into five typical activity patterns using cluster analysis. Second, we conducted multinomial logistic regression by including survey year and key covariates interactions to examine shifts between sociodemographic characteristics and activity pattern. Findings: We identified five distinct activity patterns including versatile (involves diverse types of activities), housework (daytime occupied by housework activities), work-centered (daytime dominated by paid work), leisure-intensive (long time spent in social and leisure activities), and inactive (intensive TV watching). Our findings show that SES does not affect predicted membership of the versatile group in pre-pandemic time, while an educational gradient becomes salient in 2020. Highly educated older adults are more likely to maintain the diversity of daily activities. Furthermore, the racial difference is more pronounced in the inactive group during the COVID-19 pandemic.

**Conclusion:**

Our findings suggest a buffering effect of social advantages on the impact of public health crisis.

